# Cytoplasmic Prep1 Interacts with 4EHP Inhibiting *Hoxb4* Translation

**DOI:** 10.1371/journal.pone.0005213

**Published:** 2009-04-13

**Authors:** J. Carlos Villaescusa, Claudia Buratti, Dmitry Penkov, Lisa Mathiasen, Jesús Planagumà, Elisabetta Ferretti, Francesco Blasi

**Affiliations:** 1 IFOM, FIRC Institute of Molecular Oncology, Milano, Italy; 2 Department of Biomedicine, University of Bergen, Bergen, Norway; 3 Laboratory of Molecular Genetics, San Raffaele Scientific Institute and Università Vita Salute San Raffaele, Milano, Italy; Texas A&M University, United States of America

## Abstract

**Background:**

Homeobox genes are essential for embryonic patterning and cell fate determination. They are regulated mostly at the transcriptional level. In particular, Prep1 regulates *Hox* transcription in association with Pbx proteins. Despite its nuclear role as a transcription factor, Prep1 is located in the cytosol of mouse oocytes from primary to antral follicles. The homeodomain factor Bicoid (Bcd) has been shown to interact with 4EHP (eukaryotic translation initiation factor 4E homolog protein) to repress translation of *Caudal* mRNA and to drive *Drosophila* embryo development. Interestingly, Prep1 contains a putative binding motif for 4EHP, which may reflect a novel unknown function.

**Methodology/Principal Findings:**

In this paper we show by confocal microscopy and deconvolution analysis that Prep1 and 4EHP co-localize in the cytosol of growing mouse oocytes, demonstrating their interaction by co-immunoprecipitation and pull-down experiments. A functional 4EHP-binding motif present in Prep1 has been also identified by mutagenesis analysis. Moreover, Prep1 inhibits (>95%) the *in vitro* translation of a luciferase reporter mRNA fused to the *Hoxb4* 3′UTR, in the presence of 4EHP. RNA electrophoretic mobility shift assay was used to demonstrate that Prep1 binds the *Hoxb4* 3′UTR. Furthermore, conventional histology and immunohistochemistry has shown a dramatic oocyte growth failure in hypomorphic mouse *Prep1^i/i^* females, accompanied by an increased production of Hoxb4. Finally, *Hoxb4* overexpression in mouse zygotes showed a slow *in vitro* development effect.

**Conclusions:**

Prep1 has a novel cytoplasmic, 4EHP-dependent, function in the regulation of translation. Mechanistically, the Prep1-4EHP interaction might bridge the 3′UTR of *Hoxb4* mRNA to the 5′ cap structure. This is the first demonstration that a mammalian homeodomain transcription factor regulates translation, and that this function can be possibly essential for the development of female germ cells and involved in mammalian zygote development.

## Introduction

Prep1 is a homeodomain transcription factor essential during development [1]. A hypomorphic *Prep1* mutation (*Prep1^i/i^*) shows variable penetrance and expressivity in mouse, but most *Prep1^i/i^* embryos die between E17.5 and P0 [2], [3]. Despite the low (2%) level of *Prep1* expression, about 1/4 of the homozygous *Prep1^i/i^* embryos escape embryonic lethality [4] .

Prep1 and Pbx1 form stable complexes that regulate the transcription of some *Hox* genes [1], [3]–[6]. Expression of *Hox* genes is regulated not only at the transcriptional but also at the post-transcriptional level. Indeed, *Hoxb4* expression in mouse embryos is restricted by selective translation and/or degradation of its mRNA [7]. Transcriptional and translational regulation of *homeobox* genes also occurs in *Drosophila* embryos, where nuclear Bcd regulates the transcription of *Hunchback* or *Even-skipped* in the nucleus, while in the cytosol Bcd regulates the translation of *Cauda*l (*cad*) mRNA [8]–[10]. This cytosolic effect is due to the interaction with *Drosophila* 4EHP (d4EHP) through a YxxxxxxL motif [11] distinct from the consensus binding site for the eukaryotic translation initiation factor 4E (eIF4E), YxxxxLΦ (where x is any amino acid and Φ any hydrophobic residue) [12]. d4EHP binds the 5′ cap of *cad* mRNA, while Bcd binds the 3′UTR, preventing the coordinate assembly of the translational machinery [13] .

In most animal species, female gametes contain a pool of stable stored but not translated transcripts in the cytoplasm, including *Hox* mRNAs [14]–[17]. Translation of these mRNAs occurs at meiosis, upon fertilization, and during early embryo development [16], but little information is available about *Hox* translational regulation and its importance during oocyte development.

Prep1 and Pbx1 are present in the cytosol of mouse oocytes from primary to antral follicles [18]. In early zebrafish embryos, Prep1 and Pbx1 proteins are located in the cytoplasm and they translocate to the nucleus only around gastrulation [6]. So far, no information is available about any specific developmental function of cytosolic Prep1.

Since Prep1 contains a putative 4EHP-binding motif, we have studied a possible cytoplasmic function of Prep1, discovering that Prep1 is involved in a 4EHP-dependent translational regulation of at least *Hoxb4* mRNA, and concluding that this function is possibly essential for mammalian female germ cell development.

## Results

### Prep1 interacts with 4EHP

**Figure 1:**
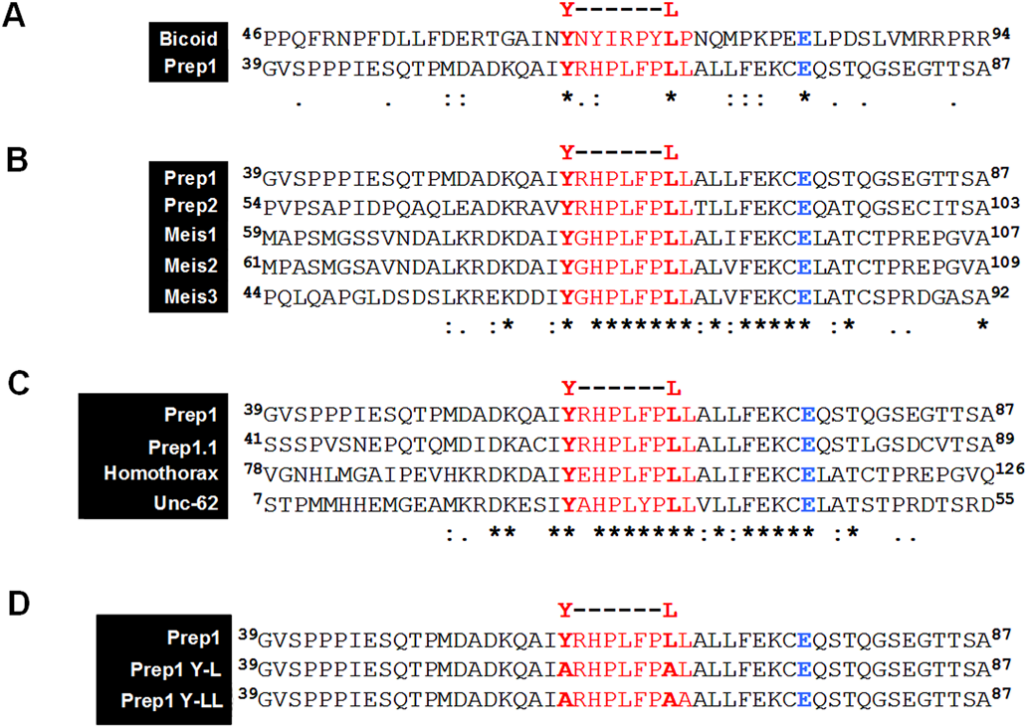
Prep1 shares a Bcd-like 4EHP-binding motif with other TALE members, and is evolutionarily conserved. (A–C) The 4EHP-binding motif is depicted in red, and the essential tyrosine (Y) and leucine (L) of the consensus domain are in red bold. Notice the conserved glutamic acid depicted in blue. (A) Sequence alignment of the amino acid region containing the 4EHP-binding motif between *Drosophila* Bcd and mouse Prep1. (B) Sequence alignment of mouse MEIS members of TALE family (Prep1, Prep2, Meis1, Meis2 and Meis3). (C) 4EHP-binding motif is evolutionarily conserved between orthologs: Prep1 (*Mus musculus*), Prep1.1 (*Danio rerio*), Homothorax (*Drosophila melanogaster*), Unc-62 (*Caenorhabditis elegans*). (D) Sequence alignment of the mutants (Y-L and Y-LL). Notice the substitution by alanines.

The ^59^ **Y**RHPLFP**LL** ^67^ amino acid motif of Prep1 (**Fig. 1A**) is similar to the ^66^ **Y**NYIRPY**L** ^73^ sequence of Bcd, that binds the translation inhibitor 4EHP [11]. This motif is present in all members of the MEIS subfamily of TALE proteins (**Fig. 1B**), and is conserved among orthologs (**Fig. 1C**). Interestingly, a glutamic acid (depicted in blue) present in Bcd is conserved in all the MEIS subfamily members. The presence of this motif and the Prep1 cytoplasmic localization in mouse oocytes [18] led us to study the interaction between Prep1 and 4EHP.

**Figure 2:**
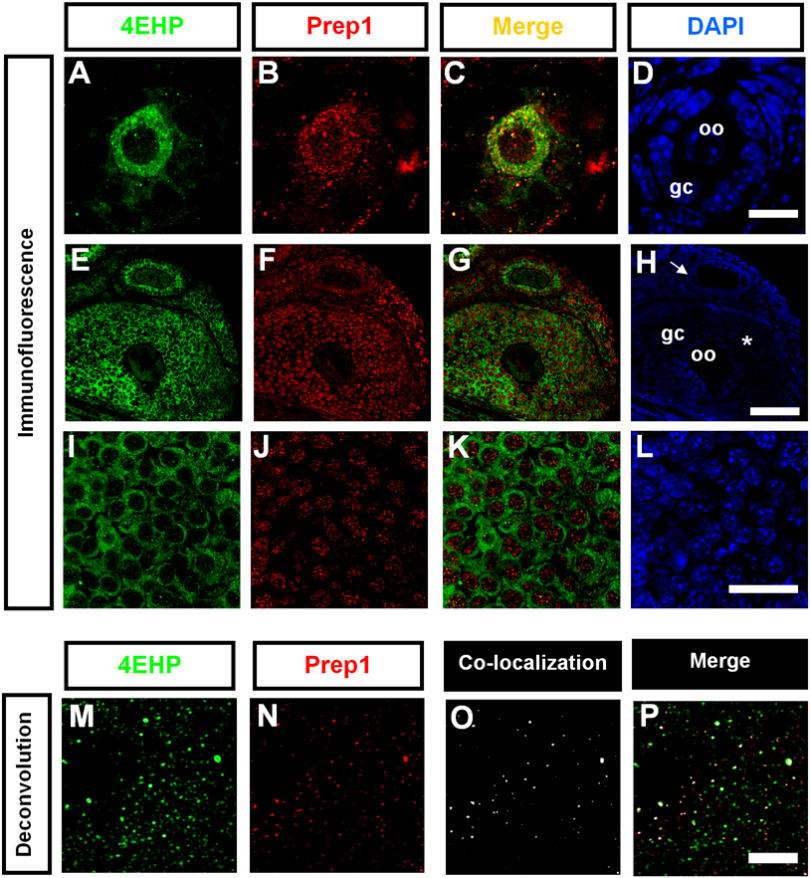
Immunofluorescence and deconvolution analysis of Prep1 and 4EHP expression in mouse ovarian follicles. (A–D) A primary follicle. The cuboidal shape of the sourrounding granulosa cells (gc) indicates the activation of the follicle. 4EHP and Prep1 are both located in the cytosol of the oocyte (oo) and colocalize (Merge, C). (E–H) Secondary (arrow) and antral (asterisk) follicles show Prep1 expression in the nucleus of granulosa cells. In contrast, 4EHP is always cytosolic and no co-localization is evident (panel G, Merge). (I–L) Granulosa cells from an antral follicle showing cytosolic localization of 4EHP. In contrast, Prep1 was clearly localized into the nucleus of the cells. Notice the absence of co-localization in the cytosol (Merge, K). (M–P) Deconvolution analysis of Prep1-4EHP localization in the cytosol of an antral oocyte. 3D co-localization analyses of 4EHP and Prep1 were performed on a voxel-to-voxel basis using automatic threshold co-localization algorithm by Costes and Locket. The image stacks obtained by confocal microscopy were deconvolved with 20 iterations using theoretical point spread function and maximum likelihood estimation algorithms of Huygens Essential software (see Materials and Methods). Notice the co-localization in white (O–P). Sale bars, D 10 µm; H 25 µm; L 15 µm; P 5 µm.

Prep1 and 4EHP co-localize in the cytoplasm of mouse primary oocytes (oo), as shown by confocal immunofluorescence analysis (**Fig. 2A–D**). From secondary to antral follicles, Prep1 is located in the nucleus of granulosa cells (gc), where 4EHP is mainly cytosolic, and no co-localization is observed (**Fig. 2E–L**). In contrast, Prep1 and 4EHP still co-localize in the cytosol of oocytes from secondary to antral follicles. The co-localization between Prep1 and 4EHP in the cytosol of antral oocytes is confirmed (**Fig. 2M–P**) by deconvolution analysis, which increases image resolution and decreases false positives [19]. As it is shown in **Fig. S1A** **,** the 4EHP antibody specifically detects 4EHP but not its close homolog eIF4E. In the case of Prep1 antibody, its specificity has been described previously [2], [3].

**Figure 3:**
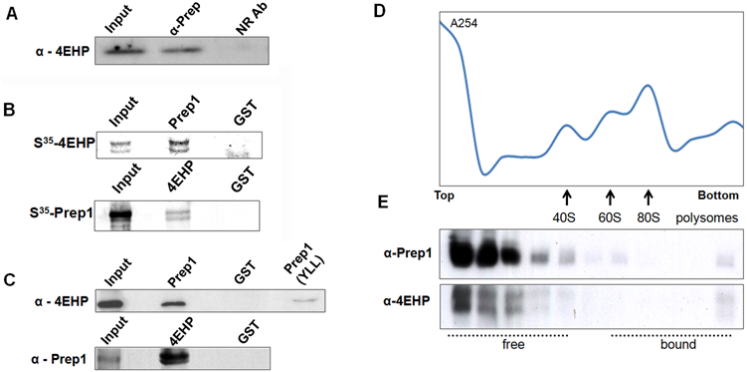
Prep1 and 4EHP interact *in vitro* and *in vivo*. (A) Anti-Prep1 antibody beads (alpha-Prep) precipitate endogenous 4EHP from cytoplasmic ovarian extracts. A non related antibody was used as a negative control (NR). (B) Prep1-GST and 4EHP-GST fusion protein beads pull-down *in vitro*-translated S^35^-Prep1 (lower) and S^35^-4EHP (upper), respectively. GST beads are used as negative control. Notice that 10% of the input was loaded in the upper part, while 50% of the input was loaded in the lower part. (C) 4EHP-GST (4EHP, upper part) and Prep1-GST fusion protein beads (Prep1, lower part) pull-down endogenous Prep1 and 4EHP from cytosolic ovarian extracts, respectively. Pulled down proteins were immunoblotted with anti-4EHP or anti-Prep1 antibodies. Notice the reduced capacity of Prep1(Y-LL)-GST beads to pull-down endogenous 4EHP. GST alone was used as a negative control. Notice that 10% of the input was loaded in the upper part, while 50% of the input was loaded in the lower part. (D) Polysome profile was analysed for cytosolic ovarian extracts by sedimentation through 15–45% sucrose gradient. (E) Prep1 and 4EHP are found in the top of the sucrose gradient fractions from a continuous 15–45% sucrose gradient. Prep1 and 4EHP were identified by immunoblotting analysis. Both Prep1 and 4EHP are found only in the first fractions, corresponding to the non ribosome associated fractions.

Prep1-4EHP interaction in ovarian cytosolic extracts was confirmed by co-immunoprecipitation of endogenous 4EHP by an anti-Prep1 antibody (**Fig. 3A**). However, the 4EHP antibody did not co-immunoprecipitate endogenous Prep1 due to the very low cytosolic Prep1 concentration and/or to the scarce immunoprecipitation capacity of the anti-4EHP antibody (data not shown). We have also investigated if Prep1-4EHP interaction is RNA mediated, but we did not observe any difference with or without RNase A treatment (data not shown). This result suggests that Prep1-4EHP interaction is not RNA mediated.

We further investigated Prep1-4EHP interaction by pulling down *in vitro* synthesized ^35^S-Met-labeled proteins. Prep1-GST and 4EHP-GST beads pulled down ^35^S-Met-4EHP and, respectively, ^35^S-Met-Prep1 (**Fig. 3B**). Moreover, 4EHP-GST or Prep1-GST beads were able to pull down endogenous Prep1 or 4EHP from ovarian cytosolic extracts, respectively (**Fig. 3C** **)**. We observed a doublet for 4EHP when it was produced *in vitro*, probably due to premature translation terminations. In contrast, a single band was observed for endogenous 4EHP (**Fig 3A–C**).

We exploited the above technique to identify the 4EHP-binding sequence in Prep1. Mutational analysis of Prep1 showed that the substitution of the conserved tyrosine 59 and leucine 66 residues with alanine (Y59A and L66A, GST-Prep1 Y-L mutant, **Fig. 1D** **)** slightly reduced the interaction between mutant Prep1 and 4EHP (data not shown). In contrast, alanine-substitution of Y59 and both L66 and L67 in Prep1 (GST-Prep1 Y-LL mutant, **Fig. 1D**) strongly reduced the interaction, even if it was not completely abolished (**Fig. 3C**).

Overall, the results show that Prep1 and 4EHP interact *in vivo* and *in vitro* and that the ^59^ **Y**RHPLFP**LL** ^67^ amino acid motif of Prep1 is functional and required for 4EHP-binding.

Prep1 protein is associated with a ribosome-free fraction of mouse ovarian cytosol

RNA-binding proteins and mRNAs are fractionated in polysomes, ribosomes and ribosome-free fractions by continuos (15–45%) sucrose gradient centrifugation [20]. In mouse ovarian post-nuclear supernatants, Prep1 and 4EHP were found in the first fractions, which do not contain ribosomes or polysomes [20], as assessed by immunoblotting (**Fig. 3D–E**). Then, we conclude that Prep1 and 4EHP are not associated with polysomal fractions.

### Prep1 co-immunoprecipitates *Hoxb4* mRNA

Bicoid homologs have been identified only in close relatives of the schizophoran fly *Drosophila*. *Stauber et al.* have shown that Bcd gene originated from a recent duplication of the direct homolog of the vertebrate gene *Hox3*, termed *zerknüllt* [21]. Prep1 is not a Hox protein, but belongs to the TALE family of homeodomain proteins, regulating *Hox* expression at the transcriptional level. For this reason, we decided to investigate if Prep1 could also regulate *Hox* genes during translation.

**Figure 4:**
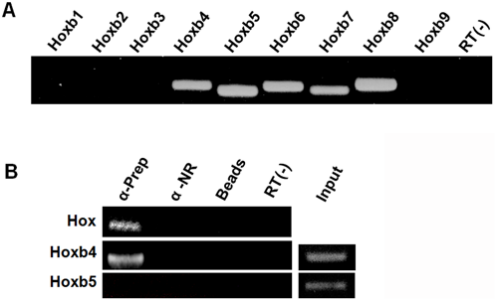
*Hoxb* cluster expression in oocytes and associated granulosa cells (OGC) and *Hoxb* mRNA immunoprecipitation by anti-Prep1 antibodies. (A) Expression of *Hoxb* genes in OGC cells by RT-PCR analysis. Notice that 5 different *Hoxb* genes (*Hoxb4–8*) are expressed. (B) Extracts from crosslinked ovarian cells (see Materials and Methods) were immunoprecipitated with anti-Prep1 or not related (NR) antibodies. The RNA was extracted and subjected to RT-PCR with degenerated *Antennapedia* primers (upper part), which amplified *Hox* messengers (*HoxA* and *B* clusters). After cloning and sequencing of the amplicons, *Hoxb4* was highly represented among the amplicons. Then, specific *Hoxb4* primers were used to confirm the previous result (middle part), amplifying *Hoxb4* mRNA from the OGC co-immunoprecipitated RNA. Notice that specific primers for *Hoxb5*, which is expressed in OGC but was not identified among the *Hox* amplicons, is not amplified from the OGC co-immunoprecipitated RNA (lower part).

RT-PCR analysis with specific primers shows that *Hoxb4*, *5*, *6*, *7* and *8* are expressed in the oocyte and associated ganulosa cells (OGC, **Fig. 4A**). To test whether Prep1 binds mRNAs coding for *Hox* genes, we immunoprecipitated crosslinked RNA from OGC using a Prep1 antibody (see Material and methods). Degenerated primers (HoxA and HoxB) based on an early nucleotide consensus for vertebrate *Antennapedia* class homeodomains [18], [22], [23] (see Materials section) were used to amplify homeobox sequences in the co-immunoprecipitated RNA from OGC. As shown in the top line of **Fig. 4B** **,** *Hox* amplicons were detected by PCR, meaning that *Hox* RNAs were co-immunoprecipitated by Prep1. After cloning and sequencing those amplicons, we found *Hoxb4* and *Hoxb8* sequences highly represented among the different clones. Knowing that *Hoxb4* and *Hoxb8* mRNAs can be co-immunoprecipitated by Prep1, we used specific primers to confirm this result. In fact, we were able to amplify *Hoxb4* and *Hoxb8* from the co-immunoprecipitated OGC RNA (**Fig. 4B** **,** second line, and data not shown for Hoxb8). In contrast, we could not amplify other Hox members from the co-immunoprecipitated RNA, such as *Hoxb5* (third line, **Fig. 4B**), which was present in OGC extracts (**Fig. 4A**). Prep1, therefore, associates at least to *Hoxb4* and *Hoxb8* mRNA in oocyte- associated granulosa cells.

### Prep1 and 4EHP co-regulate Luc-3′UTR *Hoxb4* translation *in vitro*

Since Prep1 associates with 4EHP and at least two mRNAs, these two interactions might be functionally linked. We decided to focus our work in a single mRNA, and we selected *Hoxb4* for our studies.

It has been already described that *Drosophila* and human 4EHP are able to bind cap analogs using an m^7^GTP-Sepharose approach [11], [24], [25]. For this reason, we investigated if mouse 4EHP had the same capacity. Pull-down of cytosolic extracts with m^7^GTP-Sepharose suggests that 4EHP can bind the m^7^GpppN (where N is the first template-encoded nucleotide of the transcript) cap structure of mRNAs. Both *in vitro*-translated and endogenous cytoplasmic 4EHP interact with m^7^GTP-Sepharose, but not with GTP-Sepharose (**Fig. S1B–C**). However, Prep1 does not bind m^7^GTP-Sepharose directly, as expected (not shown).

**Figure 5:**
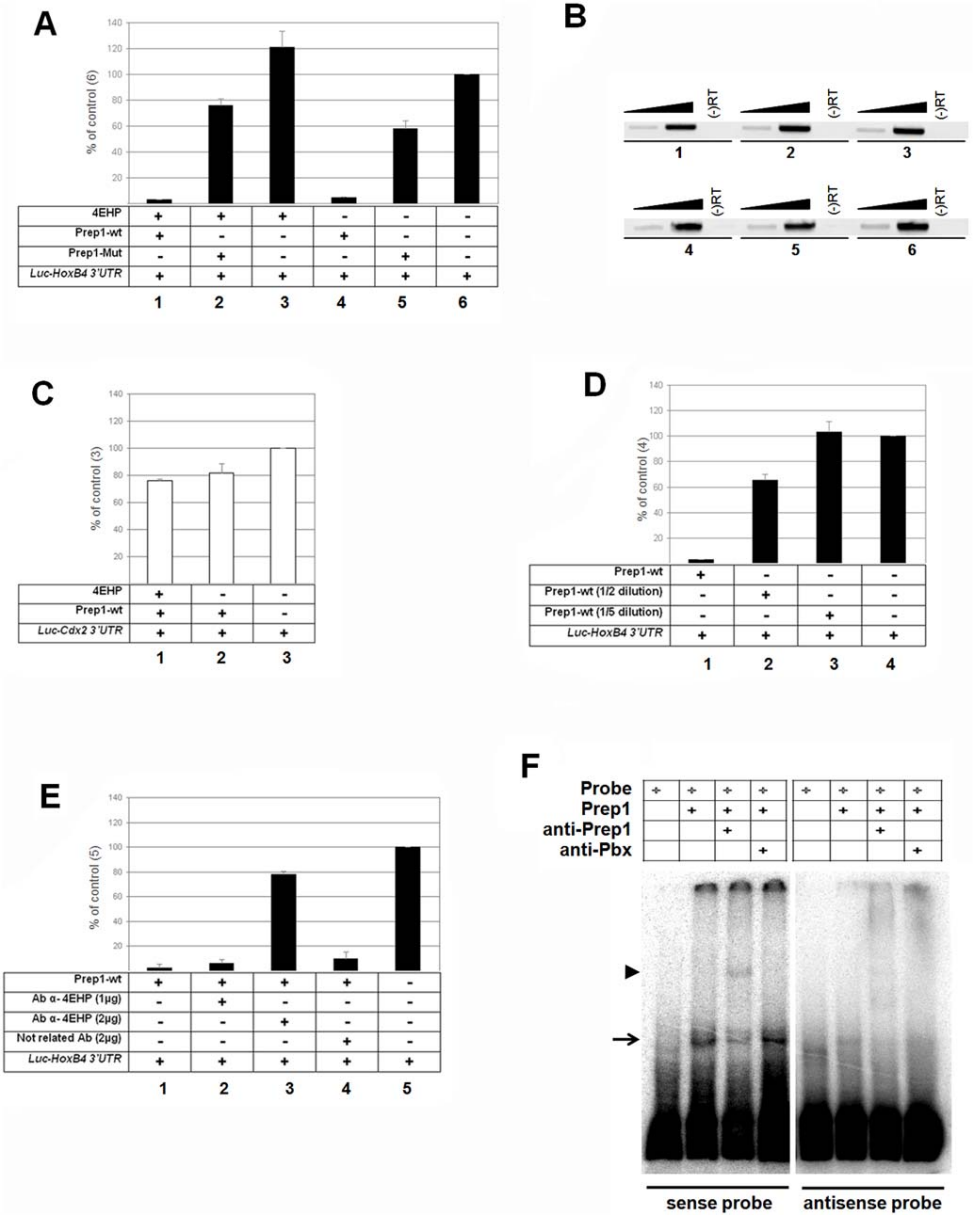
Prep1 inhibits translation of the luciferase-*Hoxb4* mRNA and is able to bind the 3′-UTR region of *Hoxb4* mRNA. (A) *Luc-Hoxb4* 3′UTR mRNA was translated *in vitro* in a rabbit reticulocytes system with the additions indicated at the bottom (Prep1 or 4EHP previously *in vitro* synthesized under T7 promoter). Luciferase activity is expressed in percent of the control; the 100% value is the level of luciferase translated in the absence of any added protein (column 6). Addition of *in vitro* translated Prep1 inhibits *Luc-Hoxb4* 3′UTR mRNA translation (columns 1 and 4) while no further effect is observed when the *in vitro* translated 4EHP protein is added to the reaction with Prep1 (column 1) or alone (column 3). Less inhibition is obtained with the *in vitro* translated Prep1 4EHP-binding (Y-LL) mutant (Prep1-Mut) (columns 2 and 5). N-values are 5. (B) This control shows that the differences observed in (A) are not due to interference with the *in vitro* transcription of the luciferase-*Hoxb4* 3′UTR mRNA. RT-PCR analysis of *Luc-Hoxb4* 3′UTR mRNA present in samples 1–6 (A). Each reaction was amplified for 25 and 30 cycles. Notice the absence of amplification in the RT(-), indicating that the plasmid used for *Luc-Hoxb4* 3′UTR transcription had been completely digested by the DNAse treatment. (C) Prep1 does not inhibit translation of *Luc-Cdx2* 3′UTR mRNA, independently of the presence of 4EHP. Thus the inhibitory effect appears to be dictated by the presence of the *Hoxb4* 3′UTR. N-values are 3. (D) Prep1 inhibits *in vitro* translation of *Luc-Hoxb4* 3′UTR mRNA in a dose-dependent manner. Compare non diluted Prep1 (column 1) with dilutions 1/2 and 1/5 (columns 2 and 3). N-values are 4. (E) Anti-4EHP antibodies prevent the inhibitory action of Prep1. Inhibition of *Luc-Hoxb4* 3′UTR mRNA translation by Prep1 was reversed when 2 µg of anti-4EHP (but not an unrelated) antibody was added to the reaction. N-values are 3. (F) RNA-EMSA showing specific Prep1 binding to *Hoxb4* 3′UTR. First lane shows the [alpha-32P] rUTP-labelled probe. Second lane shows the shift induced by the addition of Prep1 to the reaction (arrow). Third lane shows the induction of a super-shift by the Prep1 antibody (arrowhead). Lane 4 shows that the effect of the antibody is specific since an anti-Pbx antibody has no effect. Same experiment using antisense probe is shown in lanes 5–8.

Since Prep1 can bind both some mRNAs and the 4EHP translation inhibitor, we studied the effect of the Prep1-4EHP complex on *Hoxb4* mRNA translation *in vitro* using a rabbit reticulocytes lysate translation system. We cloned the 3′UTR of *Hoxb4* at the 3′ end of a luciferase reporter gene, expressed under the SP6 promoter (Luc-3′*Hoxb4*). As shown in **Fig. 5A** (n-values = 5), addition of *in vitro*-translated Prep1 (previously synthesized under the T7 promoter) inhibited Luc-3′*Hoxb4* translation by more than 90% (column 1 versus 6). In contrast, the Prep1 mutant (Prep1 YLL) inhibited only around 40% (column 2). This result completely agrees with the capacity of Prep1-YLL to bind 4EHP, which is low but not completely abolished (**Fig. 3C**). Addition of exogenous 4EHP to the reaction apparently had no major effect on Luc-3′*Hoxb4* mRNA translation (see columns 1, 4, **Fig. 5A**). However, we suspected that 4EHP may already be present in excess in the rabbit reticulocyte lysate. In fact, western blot analysis identified 4EHP in rabbit reticulocyte lysates (data not shown). Moreover, RT-PCR identified 4EHP mRNA in the micrococcal nuclease-untreated rabbit reticulocyte lysate (**Fig. S1E**) . To verify that the inhibitory effect of Prep1 was not due to a difference in the amount of RNA produced in the reaction, we also extracted total RNA from the samples shown in **Fig. 5A** and analysed the amount of Luc-3′*Hoxb4* mRNA by RT-PCR (at 25 and 30 cycles). The amount of Luc-3′*Hoxb4* mRNA produced in each reaction was comparable in all cases in non saturated PCR cycles (**Fig. 5B**), suggesting that the strong differences observed in Fig. 5A cannot be explained by a differential RNA production between reactions. Moreover, the amount of Prep1 or Prep1-YLL protein added to the reactions was comparable (**Fig. S1D**). We also verified that the inhibitory effect of Prep1 was specific for *Hoxb4* 3′-UTR. Indeed, translation of a Luc-3′*Cdx2* mRNA, containing the 3′UTR of the mammalian ortholog of *Caudal Cdx2* [26], was only marginally affected by Prep1 (**Fig. 5C** **,** n = 3). Finally, we also show that the inhibition of Luc-3′*Hoxb4* mRNA by Prep1 is dose-dependent (compare columns 1, 2 and 3 with column 4 on **Fig. 5D** **,** n = 4).

In order to address if 4EHP was required for the inhibition of Luc-3′*Hoxb4* mRNA translation by Prep1, we used a 4EHP antibody in the reaction. Addition of 2 µg of 4EHP antibody prevented the inhibition of Luc-3′*Hoxb4* mRNA translation by Prep1 (from over 95% to 20%, column 1 versus 3, **Fig. 5E** **,** n = 3). In contrast, the addition of 2 µg of an unrelated antibody (resuspended in the same buffer and at the same concentration) had no effect (column 4, **Fig. 5E** **,** n = 3).

We conclude, therefore, that Prep1 and 4EHP inhibit *in vitro* translation of mRNAs that specifically contain *Hoxb4* 3′UTR.

To identify the region of *Hoxb4* 3′UTR required for the inhibition mediated by Prep1, we subcloned *Hoxb4* 3′UTR in 3 parts (R1, R2, and R3, **Fig. S2A**) into a luciferase vector and *in vitro* translated them individually. Translation of none of the three luciferase mRNA constructs was inhibited by Prep1, suggesting that the entire 3′UTR or regions across R1-R2 or R2-R3 were necessary for Prep1 inhibition (**Fig. S2B** **,** n = 3).

Finally, we analyzed the Prep1-*Hoxb4* mRNA interaction by RNA-electrophoretic mobility shift assays using recombinant Prep1 and the *Hoxb4* 3′UTR. As a control, we used an antisense probe (**Fig. 5F**). Prep1 induced a specific mobility shift (**Fig. 5F** **,** lane 2, arrow), which was supershifted by anti-Prep1 antibody (**Fig. 5F** **,** lane 3, arrowhead). In contrast, an antibody against other transcription factors such as Pbx proteins (which recognize Pbx1, Pbx2, Pbx3 and Pbx4 members) had no effect (**Fig. 5F**, lane 4). In contrast, no binding was detected with the antisense probe. This confirms that Prep1 specifically binds *Hoxb4* 3′UTR mRNA. Whether 4EHP is required to increase Prep1 affinity for the 3′UTR has not been investigated.

### Prep1 hypomorphic mice show drastic defects in ovary and oocyte development

**Figure 6:**
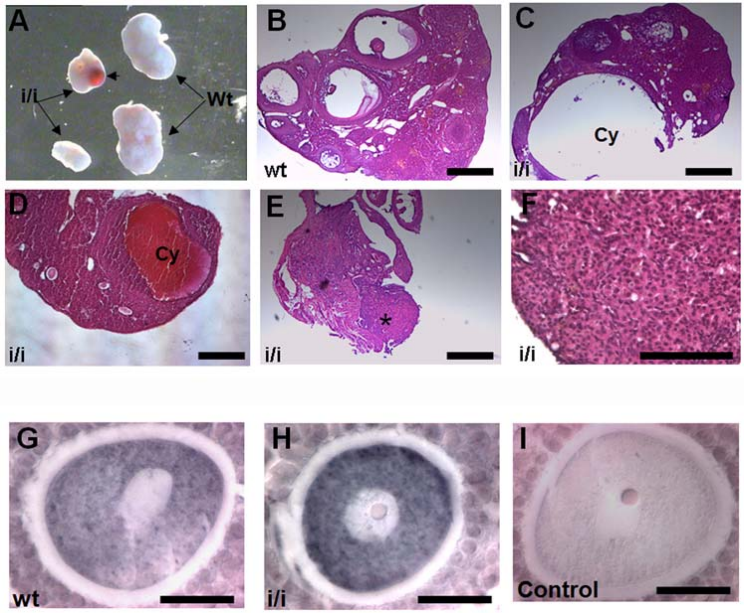
Ovarian phenotype of the *Prep1^i/i^* mice. (A) Image showing the developmental failure of *Prep1^i/i^* ovaries. Ovaries were smaller in size (compare i/i versus wt), and in almost half of the ovaries analyzed a cyst was observed (arrowhead). (B–D) Haematoxylin and eosin staining of *Prep1^i/i^* ovarian sections (C and D) showing the cyst (Cy) formation and the reduced number of follicles compared with Wt (B). Notice that most of the follicles found in *Prep1^i/i^* section (D) were primary or secondary follicles. (E) Absence of developed ovary. The asterisk marks the structure that might correspond to the undeveloped ovary. (F) Higher magnification of a non developed ovary, where no follicles were detected. (G–I) Hoxb4 immunostaining of mouse oocytes. Notice the strong staining present in the *Prep1^i/i^* oocyte compared with wild-type. The control (I) was performed without primary antibody. Scale bars, B–E 25 µm; F 15 µm; G–I 10 µm.

To test for an *in vivo* role of Prep1 in oocytes and ovary development, we analyzed some of the very few *Prep1^i/i^* females that reach adulthood [3]. Because of the low number (n = 5) of available mice, we cannot claim that homozygous *Prep1^i/i^* females are sterile, but we have never observed pregnancies in mouse *Prep1^i/i^* females. However, *Prep1^i/i^* ovaries had a drastic phenotype: they were smaller and underdeveloped (10/10), presented no oocytes (5/10) or developed cysts (4/10) (**Fig. 6A–F**).

### *Hoxb4* expression is increased in *Prep1^i/i^* oocytes

If the Prep1-4EHP interaction negatively regulates *Hoxb4* mRNA translation in mouse oocytes, one would expect an increased Hoxb4 production in *Prep1^i/i^* oocytes. Indeed, Hoxb4 was increased in *Prep1^i/i^* oocytes in about 40% of the secondary to antral oocytes (10 *Prep1^i/i^* ovaries analysed, with 16 secondary to antral oocytes in total, **Fig. 6G–I**). No differences were observed in primary follicles, where Hoxb4 was almost undetectable by immunohistochemistry (data not shown). These data suggest a translation-inhibition function of cytosolic Prep1 *in vivo,* and indicate that Prep1 could repress *Hoxb4* mRNA translation in oocytes. Interestingly, Hoxb4 was localised in the cytosol in antral oocytes.

### Injection of *Hoxb4* in mouse zygotes delays embryo development *in vitro*

**Figure 7:**
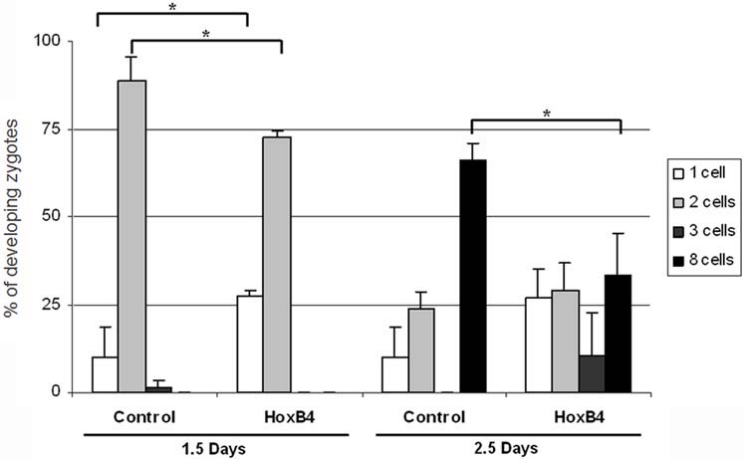
Hoxb4 overexpression in mouse zygotes delays development. Distribution of 1, 2, 3, and 8-cell embryos at 1.5 or 2.5 days after injection of CMV-IRES-GFP vector (control) or CMV-Hoxb4-IRES-GFP vector (Hoxb4). Notice the high number of 1-cell embryos and the low number of 2-cell embryos at 1.5 days after *Hoxb4* injection, compared with control. At 2.5 days after injections, the number of embryos injected with Hoxb4 vector reaching the 8-cell stage is less than 50% of the number obtained after control vector injection. This suggests that there is a delay in early embryo development when *Hoxb4* is overexpressed. (*) P value<0.04, as determined by Student's t test.

In order to test whether the oocyte phenotype of *Prep1^i/i^* mice correlates with the increased *Hoxb4* mRNA translation, we micro-injected fertilized oocytes from super-ovulated females with either CMV-IRES-GFP or CMV-Hoxb4-IRES-GFP vector and examined their development in culture. The overall death rate due to micro-injection was not significantly different between *GFP* and *Hoxb4* injected zygotes (not shown). Those zygotes lysed within the first 24 hours were not included in the calculations. We performed three series of injections for each vector, using 140 fertilized oocytes with the control and 240 with the Hoxb4 vector. Fluorescence microscopy showed that the GFP was expressed at very low levels in several (although not all) injected zygotes, at the various stages (**Fig. S2C**). **Figure 7** shows the (averaged) results of the three experiments in which at 24 hour intervals the percentage of embryos at each developmental stage (1–2 cells and 3–8 cells) was scored and expressed as percent of the total “live” embryos. Overall, the development was slowed down at all stages in the *Hoxb4*-microinjected zygotes. The results were statistically significant at the very early (1–2 and 3–8 cells) stages. Overexpression of *Hoxb4* showed the same trend also at the morula/blastocyst stage, where it did not reach statistical significance (not shown). We conclude, therefore, that the overexpression of *Hoxb4* in mouse zygotes slows down embryo development.

## Discussion

Nuclear Prep1 transcription factor forms a ternary complex with Pbx1 and Hoxb1, which is required for *Hoxb1* and *Hoxb2* transcription in embryonic rhombomeres 4, 6 and 7, respectively [3], [6], [27], [28]. However, the possible function of cytosolic Prep1 and Pbx1 is not known [6], [18].

In *Drosophila* embryos, the homeodomain protein Bcd interacts with 4EHP to regulate the translation of *Cad* mRNA through a YxxxxxxL motif [11]. Although Bcd homologs have been identified only in close relatives of *Drosophila*, we show in this paper that the ability to act in both transcriptional and translational levels is conserved in some mammalian homeodomain proteins, and that at least the TALE class protein Prep1 specifically represses translation of *Hoxb4* mRNA.

Bcd and Prep1 mechanisms are different. First, Bcd is related to Hox [21], not to TALE proteins. Second, cytosolic Bcd regulates embryonic patterning while cytosolic Prep1 in mammals likely regulates *Hoxb4* expression in female germ cells. Moreover, Bcd represses *Cad* mRNA during embryo development, but we were not able to find any apparent effect of Prep1 on *Cdx2* 3′UTR mRNA (the mammalian ortholog of *Cad*). Another difference lies at the level of the 4EHP-interacting sequence which is ^66^ **Y**NYIRPY**L** ^73^ in Bcd and ^59^ **Y**RHPLFP**LL** ^67^ in Prep1 (**Fig. 1A**), i.e. with an additional important leucine in the case of Prep1. The ^59^ **Y**RHPLFP**LL** ^67^ sequence present in Prep1 is highly conserved in proteins of the same family, suggesting that the translation inhibition function might be shared with other members of the family (**Fig. 1B**). If this prediction is verified, it is possible that members of the same family are able to bind different mRNAs. Interestingly, the Prep1 4EHP-binding sequence overlaps with the Pbx1-binding sequence [29], suggesting that the binding of Prep1 to 4EHP or to Pbx1 is mutually exclusive. This agrees with the ability of Prep1 to bind the 3′UTR of *Hoxb4* in the absence of any Pbx proteins. In fact, this could explain why Prep1 is located in the cytosol of mouse oocytes. The formation of a Prep1-Pbx complex is necessary to transport Prep1 to the nucleus [30].

Translation inhibition by Prep1-4EHP is most likely due to the inability of 4EHP to bind eIF4G [31]. The interaction with 4EHP-Prep1 would sequester the target mRNA preventing its association with the translation initiation machinery. Unlike Hoxa9 [12], we were not able to find an interaction between Prep1 and the translation initiation factor eIF4E (not shown).

In this paper, we have focused our study on *Hoxb4*. However, the target of Prep1 may be not only *Hoxb4* mRNA, since *Hoxb4* mRNA was not the only one co-immunoprecipitated in our experiments. Moreover, although we have not demonstrated the formation of a Prep1-mRNA-4EHP complex, Prep1 might bind simultaneously to 4EHP and to *Hoxb4* 3′UTR mRNA. In turn, mRNA would be bound by 4EHP at the cap site. The Prep1-binding sequence in *Hoxb4* mRNA is located in the 3′UTR, since Prep1 inhibition was specific for this 3′UTR, but Prep1 was unable to repress translation when only part of the 3′UTR was present (**Fig. S2B**). However, we cannot exclude that the *Hoxb4* 3′UTR binding region is located in a sequence bridging R1 to R2 or R2 to R3.

Translational control is an important mechanism regulating the earliest stages of embryogenesis [16], [32], [33]. In mammals, maternal mRNA translation is tightly controlled delaying translation of specific maternal mRNAs during the mammalian oocyte-embryo transition [34]–[36]. The novel oocyte/ovary phenotype of the *Prep1^i/i^* mice correlates with the increased production of Hoxb4. The increased synthesis of Hoxb4 protein *in Prep1^i/i^* oocytes agrees with the hypothesis that the absence of Prep1 relieves a block of *Hoxb4* mRNA translation leading to an oocyte growth failure and cyst formation. However, *Hoxb4* null mutant females are viable and fertile [37], possibly due to compensation by another *Hox* gene. On the other hand, overexpression of *Hoxb4* in mouse developing oocytes leads to developmental delay at the transition between one to eight cells, and the same trend is also observed at morula/blastocyst stages. In fact, Prep1 is the first homeodomain protein whose translational repression activity may be functionally relevant *in vivo* in mammals.

In summary, we conclude that Prep1 is involved in translational regulation of *Hoxb4* mRNA in mouse oocytes, in cooperation with 4EHP. This function may be essential for mammalian female germ cell development and also involved during the first stages of embryo development.

## Materials and Methods

### Prep-1 targeting

Prep1^i/i^ mice and embryos, as well as the PCR genotyping strategy, have been described previously [2]–[4].

### Animals

C57BL/6 3 months old female mice (Charles River, Italy) were used. Animal handling followed European Community recommendations.

### Immunoflorescence and immunohistochemistry

For immunofluorescence, deparaffinated sections (7 µm) were treated for epitope unmasking in 500 mM sodium citrate (pH 6). Then, sections were processed as described previously [2]. Anti-Prep1 antibody (Upstate) 1∶100 dilution; anti-4EHP (Abcam) 1∶50 dilution were used. For immunohistochemistry, after the primary antibody incubation (Prep1 from Upstate; Hoxb4 from Santa Cruz Biotechnologies, 1∶50 dilution), sections were processed as described previously [3].

Images were taken in a Leica TCS SP2 confocal microscope, using the acquisition software Leica Power Scan, at IFOM-IEO Campus (http://imaging.service.ifom-ieo-campus.it/index.html).

Figures in this paper were prepared using the Adobe Phostoshop CS4 version 11.0.

### Sucrose gradient

We followed the protocol described previously [20].

### RNA immunoprecipitation

We modified a protocol described previously [38]. Ovaries were dissected under the microscope, and oocytes with surrounding granulosa cells were isolated. Cells were washed twice with 5ml PBS, and resuspended in 2ml PBS. Formaldehyde was added to a final concentration of 1% and incubate at RT for 10 min with slow mixing. Reaction was quenched by the addition of glycine (pH 7.0) to a final concentration of 0.25M, followed by incubation at RT for 5min. Cells were harvested by centrifugation using a clinical centrifuge at 3000rpm for 5min. Cells were washed twice with ice-cold PBS. Fixed cells were resuspended in 2ml of IP buffer (20mM HEPES-KOH, pH 7.6, 200mM KCl, 0.5mM EDTA, 10% glycerol, 0.5% Triton X-100 and Protease Inhibitor Cocktail Complete; Roche). Cells were lysed routinely by three rounds of sonication, 30s each. Between each cycle, the samples were kept in an ice-water bath for 2min. Insoluble material wass removed by microcentrifugation at 14.000rpm for 10min at 4°C. Immunoprecipitation was performed by adding the relevant antibody to the supernatant extracts and incubating at 4°C overnight. Reactions were incubated with 20 µl protein A slurry beads (equilibrated in IP buffer containing 1mg/ml BSA, competitor tRNA at 100 µg/ml) and the mix was incubated for 2h at 4°C. Beads were collected using a minicentrifuge at 6.000rpm for 45s and the supernatant was saved for RNA extraction. Beads were washed five times with 1ml of IP buffer by 15min rotation at 4°C. Beads were collected and resuspended in 100 µl of 50mM Tris-Cl pH 7.0; 5mM EDTA; 10mM DTT and 1% SDS. Beads were incubated at 70°C for 3h to reverse crosslinks. RNA extraction was performed with Quiagen RNasin kit. After RNA extraction, a DNA digestion was performed, and RNA was cleared by the same kit.

### DNA Constructs, primers, and site-directed mutagenesis

Mouse Prep1, 4EHP and eIF4E cDNAs were cloned in our laboratory by PCR. Prep1, 4EHP were inserted in pcDNA3.1 (Invitrogen), under T7 promoter, and in pGEX6p vector (GE Healthcare). eIF4E was also inserted in pGEX6p. The Luciferase plasmid used for the luciferase assay experiments was the Luciferase SP6 Control DNA plasmid (4747bp). After cloning, every single insert was confirmed by sequencing. Primers used to amplify mouse 4EHP were the following (EcoRI restriction sites are underlined),

Forward 5′-CGGAATTCATGAACAACAAGTTCGACGC-3′

Reverse 5′-CGGAATTCTCATGGCACATTCAATCGCG-3′

For Prep1 (SalI restriction sites are underlined),

Forward 5′-ACGCGTCGACCTATGATGGCGACACAGACGCTAAG-3′

Reverse 5′-ACGCGTCGACCTACTGAAGGGAGTCGCTGTTCTCC-3′

Alanine substitutions were generated by site-directed mutagenesis according to the QuikChange protocol (Stratagene). Y59, L66 and L67 residues present in the Prep1 sequence were substituted with alanines. Primers used were the following,

For Y59,

Forward 5′-CGACAAGCAGGCCATTGCTAGGCATCCACTATTTCC-3′

Reverse 5′-GGAAATAGTGGATGCCTAGCAATGGCCTGCTTGTCG-3′

For L66,

Forward 5′-CATCCACTATTTCCGGCGCTAGCTTTGTTGTTTGAG-3′

Reverse 5′-CTCAAACAACAAAGCTAGCGCCGGAAATAGTGGATG-3′

For L67,

Forward 5′-CATCCACTATTTCCGGCGGCGGCTTTGTTGTTTGAG-3′

Reverse 5′-CTCAAACAACAAAGCCGCCGCCGGAAATAGTGGATG-3′

Primers used to clone the mouse HoxB4 3′UTR region were,

Forward 5′-CCGAGCTCTGCCCCCCAAGCAGGAGTTCG-3′

Reverse 5′-CCGAGCTCAAAGGAAGAAAGCAAGAGACT-3′

And for mouse Cdx2 3′UTR,

Forward 5′-CCGAGCTCGTGACCCCTCCCGTGGTCTG-3′

Reverse 5′-CCGAGCTCATACAACTTCTCTACCCATG-3′

Primers used to clone the 3 Hoxb4 3′UTR regions (R1, R2 and R3) were,

B41 5′-CCGAGCTCAGGGTCCCCGGGCTTGA-3′

B42 5′-CCGAGCTCAGAAGGGGGGTAGGGAA-3′

B41b 5′-CCGAGCTCTCAAGCCCGGGGACCCT-3′

B42b 5′-CCGAGCTCTTCCCTACCCCCCTTCT-3′

Primers used to amplify Hoxb cluster were (forward and reverse),

For Hoxb1,

5′-GTCAGTCGGAAGGAGATGGA-3′

5′-AGTCCCAGCTCGGACACCTTC-3′

For Hoxb2,

5′-CTCCCGATCTCAGCTAAACG-3′

5′-CTTCTCCAGCTCCAGCAGTT-3′

For Hoxb3,

5′-CCGCACCTACCAGTACCACT-3′

5′-GAACTCCTTCTCCAGCTCCAC-3′

For Hoxb4,

5′-TTCACGTGAGCACGGTAAAC-3′

5′-GTTGGGCAACTTGTGGTCTT-3′

For Hoxb5,

5′-GCAGACTCCACAGATATTCC-3′

5′-TGATCTGACGCTCGGACAGG-3′

For Hoxb6,

5′-GAGACCGAGGAGCAGAAGTG-3′

5′-AACCAAATCTTGATCTGCCG-3′

For Hoxb7,

5′-TTCCTTCAACATGCACTGCG-3′

5′-TTTCTCCAGCTCCAGGGTCT-3′

For Hoxb8,

5′-GGTGCGCAGGATCCAGACCT-3′

5′-ATACCTCGATCCTCCGCTTGC-3′

For Hoxb9,

5′-AATCAAAGAGCTGGCTACGG-3′

5′-GTCTCTCACTCAGATTGAGG-3′

### Mouse Prep1, 4EHP and eIF4E-GST Protein Production

For the purification of mouse 4EHP-GST, eIF4E-GST, and Prep1-GST fusion proteins, *E. Coli* MJ109 was transformed with pGEX6p-4EHP, pGEX6p-eIF4E, or pGEX6p-Prep1 construct (GE Healthcare). Protein expression was induced with 1mM IPTG. Expression was continued for ∼2h at 37°C. Cells were harvested by centrifugation and resuspended in 30ml lysis buffer (20mM Tris, pH 7.4, 0.3mM NaCl, 1mM DTT supplemented with Protease inhibitor cocktail from Roche) per litre of culture. After sonication, the lysate was cleared by centrifugation. The GST-fusion protein was purified using Glutathione-agarose beads (GE Healthcare) equilibrated in 20mM Tris, pH 7.4, 0.3mM NaCl, 1mM DTT.

### Cap-Affinity Assay

For Cap-affinity assay we followed the protocol described previously [11].

### Deconvolution analysis

Confocal microscopy stacks were deconvolved with 20 iterations using theoretical point spread function (PSF) and maximum likelihood estimation (MLE) algorithms of Huygens software (SVI, Hilversum, the Netherlands). 3D colocalization analyses of 4EHP and Prep1 were performed using the automatic threshold algorithm by Costes and Locket [39] implemented in Bitplane Imaris suite (Bitplane AG, Zurich, Switzerland). 3D colocalization is shown as the white channel.

### Co-immunoprecipitations and GST Pull-Down

For co-immunoprecipitation, cytosolic ovarian cell extract was brought up to 0,5 ml with the IP buffer (20 mM HEPES-KOH, pH 7.6, 200 mM KCl, 0.5 mM EDTA, 10% glycerol, 0.5% Triton X-100 and Protease Inhibitor Cocktail Complete; Roche) and precleared for 1h at 4°C with 25 µl of Protein A Sepharose. The supernatant was immunoprecipitated for 1h at 4°C with 25 µl of anti-Prep (Santa Cruz Biotech). The resin was washed three times with lysis buffer. Immunoprecipitates were eluted in 2× sample buffer.

### Immunoblotting

After denaturation, the samples were resolved in 10% SDS-PAGE and transferred electrophoretically to Nitrocellulose membranes (Amersham). Then, membranes were processed as described previously [18]. Anti-Prep1 (1∶100, Upstate); anti-4EHP (1∶500, Abcam).

### Luciferase Assay

Prep1, mutant-Prep1 and 4EHP proteins were generated using the TNT T7 Coupled Reticulosyte Lysate Transcription/Translation System (Promega), under the T7 promoter following the manufacturer's instructions. As a control-reaction, a T7-reaction with an empty pCDNA3.1 vector was used. T7-reactions were stopped on ice after 1h of incubation at 30°C. 1 µl of the corresponding T7-reactions (containing Prep1, mutant-Prep1, 4EHP protein or control-reaction) was added to the SP6-reactions composed by 20 µl of master-mix, methionine, Luc-*Hoxb4* 3′UTR plasmid (or SP6 Luciferase vector with the R regions of *Hoxb4* 3′UTR), and SP6 enzyme, in a total volume of 25 µl following the manufacturer's instructions. SP6-reactions were incubated at 30°C for 1h and 30min. After the 1^st^ hour of incubation, the reaction was shacked vigorously for 5 seconds. Reactions were stopped on ice. Then, 2.5 µl were used to analyze luciferase production.

SP6-Reactions were peformed everytime in triplicate, and each condition was performed at least three independent times (see n-values in the text).

### mRNA extraction and RT-PCR

mRNA extraction from luciferase samples were extracted, and retrotranscribed as previously described [18].

We also extracted total RNA from fresh non treated lysate (Promega, Rabbit Reticulocyte Lysate, Untreated, cat. L4151).

Degenerate primers for amplification of HoxA and HoxB cDNA were used as described previously [18], [22], [23]. Amplified products were cloned with TA-Cloning kit (Invitrogen), sequenced, and screened for homology to known sequences using the NCBI-BLAST software.

### Cloning, expression and purification of human Prep1

Human Prep1 protein was used just only for REMSA experiments. Expression in the *Escherichia coli* strain BL21(DE3) was induced with 0.3mM IPTG. Expression was continued for ∼16h at 20°C. Cells were harvested by centrifugation and resuspended in 30ml lysis buffer (20mM Tris, pH 7.4, 0.3mM NaCl, 1mM DTT supplemented with Protease inhibitor cocktail from Calbiochem) per litre of culture. After sonication, the lysate was cleared by centrifugation. The GST-fusion protein was purified using Glutathione-agarose beads (GE Healthcare) equilibrated in 20mM Tris, pH 7.4, 0.3mM NaCl, 1mM DTT and the protein was subsequently cleaved from GST with 10u of PreScission protease (GE Healthcare) per milligram of substrate for 16h at 4°C.

### REMSA

The probe for REMSA was prepared and labeled by the in vitro transcription of the cloned DNA fragment of Hoxb4 3′UTR using [alpha-32P]rUTP and Riboprobe® Combination System (Promega, Madison, WI). After treatment with DNase, it was described by RNeasy kit (Qiagen, Germany). REMSA was carried out as previously published with minor modifications [40]. Briefly, the reaction was performed in the CEB buffer (10mM HEPES, pH 7.5, 3mM MgCl2, 14mM KCl, 5% glycerol, 1mM DTT) using 0.3 µg of human Prep1 recombinant protein. After 20min incubation on ice with or without 5 µg of anti-Prep1 [30] or anti-Pbx1 (sc-889X, Santa-Cruz Biotechnology, Santa Cruz, CA), the probe (50,000 cpm) was added and the mixture incubated at room temperature, followed by 10min incubation with RNase T1 (0.5u) and 10min incubation with heparin (6mg/ml). The RNA-protein complexes were resolved in 5% polyacrlylamide mini-gels (acrylamide:bis acrylamide of 36∶1) and vacuum-dried. RNA-protein interactions were visualized by use of PhosphoImager 445 SI (Molecular Dynamics Sunnyvale, CA).

### Microinjection of Hoxb4 into mouse oocytes

Fully grown, germinal vesicle-intact (GV) fertilized mouse oocytes were obtained from 4-week-old female mice and freed of attached cumulus cells as previously described [41], [42]. The collection medium was bicarbonate-free minimal essential medium (Earle's salts) supplemented with polyvinylpyrrolidone (3mg/ml) and 25mM Hepes, pH 7.3. The denuded oocytes were matured in CZB medium [43] in an atmosphere of 5% CO2 in air at 37°C. Images were captured by Zeiss Discovery V12 stereo microscope, and fluorescence with Nikon SMZ 1500 Microscope.

### Statistical analysis

All the experiments were performed at least three times. For statistical analysis of data, Student's t test was used. Values are expressed as mean±standard error of the mean. Data were considered statistically different at a *p* value of <0.04.

## Supporting Information

Figure S1 (A) This control shows the specificity of the anti-4EHP antibody that does not recognize the close homolog eIF4E. (B) Cytosolic extracts from wild type mouse ovaries were pulled down using m7-GTP or GTP (control) beads and eluted as described in the Material and Methods section. The presence of 4EHP in the eluate was monitored by immunoblotting. (C) Same experiment as in (B), but performed with in vitro translated 35S-4EHP. (D) This control shows that the amounts of Prep1 and mutant Prep1 added to the reactions (Fig. 5A) were equivalent, as shown by the radiographic evaluation of in vitro translated 35S-Met-labeled proteins. (E) 4EHP messenger RNA is detected in the crude untreated rabbit retyculosyte lysate, suggesting that there is at least endogenous 4EHP mRNA in the reaction.
(2.76 MB TIF)

Figure S2 (A) Hoxb4 mRNA sequence, from the stop codon TAG (black box) to the poly-A signal. The Hoxb4 3′UTR was divided in 3 regions (R1, R2, and R3) and cloned using specific primers (sequences underlined) in a luciferase vector, in order to study the effect of Prep1 protein. (B) Prep1 does not inhibit the translation of luciferase-Hoxb4 R1, R2 or R3 3′UTR mRNA, suggesting that the whole 3′UTR is required for the inhibition. (C) Expression of fluorescent GFP in mouse embryos micro-injected with a CMV-Hoxb4-IRES-GFP construct (mouse embryos, left; GFP merge, right). This representative picture was taken at an early developmental stage, after 1.5 days in culture.
(5.48 MB TIF)
