# Supplementary figures and images for: Cytoplasmic Prep1 Interacts with 4EHP Inhibiting Hoxb4 Translation
Source: PLoS One. 2009 Apr 13;4(4):e5213. doi: 10.1371/journal.pone.0005213 (PMC2664923; doi:10.1371/journal.pone.0005213)

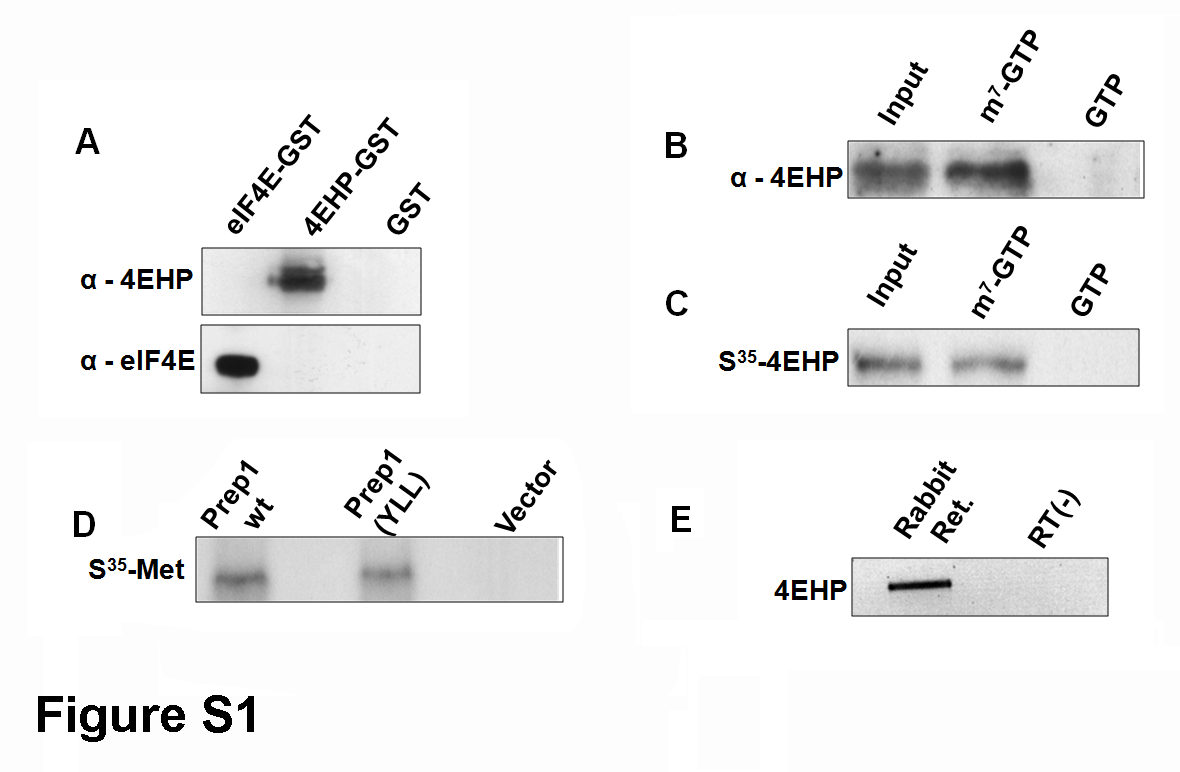

Supplement: Figure S1 — (A) This control shows the specificity of the anti-4EHP antibody that does not recognize the close homolog eIF4E. (B) Cytosolic extracts from wild type mouse ovaries were pulled down using m7-GTP or GTP (control) beads and eluted as described in the Material and Methods section. The presence of 4EHP in the eluate was monitored by immunoblotting. (C) Same experiment as in (B), but performed with in vitro translated 35S-4EHP. (D) This control shows that the amounts of Prep1 and mutant Prep1 added to the reactions (Fig. 5A) were equivalent, as shown by the radiographic evaluation of in vitro translated 35S-Met-labeled proteins. (E) 4EHP messenger RNA is detected in the crude untreated rabbit retyculosyte lysate, suggesting that there is at least endogenous 4EHP mRNA in the reaction. (2.76 MB TIF) [file pone.0005213.s001.tif]

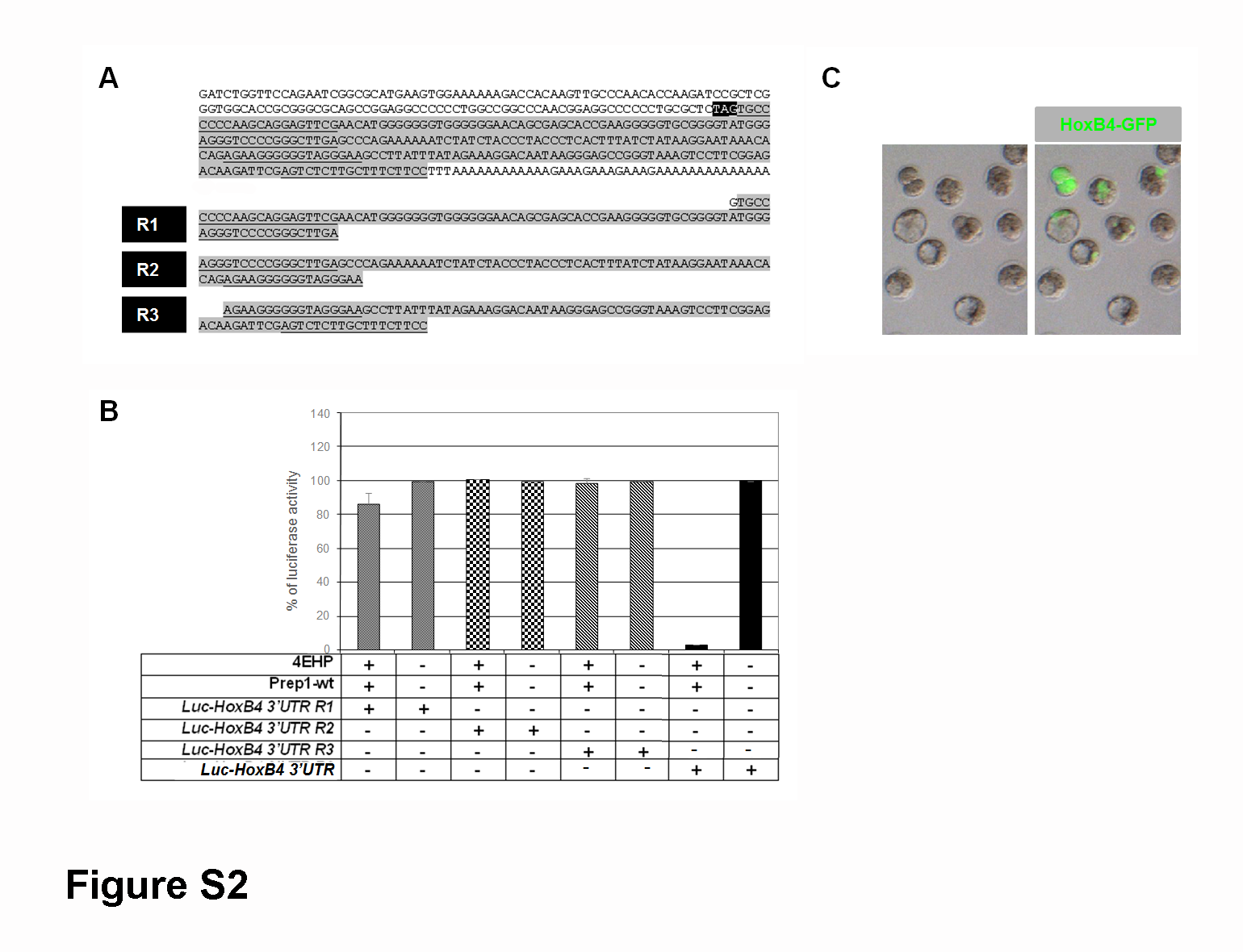

Supplement: Figure S2 — (A) Hoxb4 mRNA sequence, from the stop codon TAG (black box) to the poly-A signal. The Hoxb4 3′UTR was divided in 3 regions (R1, R2, and R3) and cloned using specific primers (sequences underlined) in a luciferase vector, in order to study the effect of Prep1 protein. (B) Prep1 does not inhibit the translation of luciferase-Hoxb4 R1, R2 or R3 3′UTR mRNA, suggesting that the whole 3′UTR is required for the inhibition. (C) Expression of fluorescent GFP in mouse embryos micro-injected with a CMV-Hoxb4-IRES-GFP construct (mouse embryos, left; GFP merge, right). This representative picture was taken at an early developmental stage, after 1.5 days in culture. (5.48 MB TIF) [file pone.0005213.s002.tif]
